# KAT8 acetylation-controlled lipolysis affects the invasive and migratory potential of colorectal cancer cells

**DOI:** 10.1038/s41419-023-05582-w

**Published:** 2023-02-27

**Authors:** Bingquan Qiu, Shen Li, Meiting Li, Shuo Wang, Guanqun Mu, Keyu Chen, Meng Wang, Wei-guo Zhu, Weibin Wang, Jiadong Wang, Ziyu Li, Jichun Yang, Yang Yang

**Affiliations:** 1 Beijing Key Laboratory of Protein Posttranslational Modifications and Cell Function, Department of Biochemistry and Biophysics, School of Basic Medical Sciences, Peking University Health Science Center, 38 Xueyuan Road, Beijing, 100191 China; 2 Key Laboratory of Carcinogenesis and Translational Research (Ministry of Education), Department of Gastrointestinal Surgery, Peking University Cancer Hospital & Institute, Beijing, 100142 China; 3 Department of Biochemistry and Molecular Biology, Shenzhen University School of Medicine, 518055 Shenzhen, China; 4 Department of Radiation Medicine, Institute of Systems Biomedicine, School of Basic Medical Sciences, Peking University Health Science Center, Beijing, 100191 China; 5 Department of Physiology and Pathophysiology, School of Basic Medical Sciences, Key Laboratory of Molecular Cardiovascular Science of the Ministry of Education, Center for Non-coding RNA Medicine, Peking University Health Science Center, Beijing, 100191 China

**Keywords:** Acetylation, Epigenetics

## Abstract

Epigenetic mechanisms involved in gene expression play an essential role in various cellular processes, including lipid metabolism. Lysine acetyltransferase 8 (KAT8), a histone acetyltransferase, has been reported to mediate de novo lipogenesis by acetylating fatty acid synthase. However, the effect of KAT8 on lipolysis is unclear. Here, we report a novel mechanism of KAT8 on lipolysis involving in its acetylation by general control non-repressed protein 5 (GCN5) and its deacetylation by Sirtuin 6 (SIRT6). KAT8 acetylation at K168/175 residues attenuates the binding activity of KAT8 and inhibits the recruitment of RNA pol II to the promoter region of the lipolysis-related genes *adipose triglyceride lipase* (*ATGL*) and *hormone-sensitive lipase* (*HSL*), subsequently down-regulating lipolysis to affect the invasive and migratory potential of colorectal cancer cells. Our findings uncover a novel mechanism that KAT8 acetylation-controlled lipolysis affects invasive and migratory potential in colorectal cancer cells.

## Introduction

Emerging evidence shows the wide interplays between epigenetic regulation and cell metabolism [1, 2]. Colorectal cancer (CRC) is one of the most common malignant diseases with a high mortality rate characteristic of metabolism disorder, including lipid metabolism [3–6]. Recently, some studies revealed that histone deacetylases played key roles in lipid metabolism of colorectal cancer [7, 8]. For example, bouchardatine was reported to suppress rectal cancer by disrupting its metabolic pathways *via* activating the SIRT1-PGC-1α-UCP2 axis [8]. In addition, our previous data showed that SIRT6 is phosphorylated by PKCζ at threonine 294 residue, which promotes SIRT6 enrichment on the chromatin to regulate the expression of fatty acid β-oxidation-related genes [9]. Also, we found that p53 physically interacts with histone deacetylase SIRT6 in vitro and in vivo, and cooperates with SIRT6 to regulate cardiolipin de novo biosynthesis [10]. However, the function of histone acetyltransferase in lipid metabolism of colorectal cancer is seldom mentioned and needs to further explore.

Lysine acetyltransferase 8 (KAT8), also called MOF (Males Absent on the First), is a histone acetyltransferase that belongs to MYST family. KAT8 is a chromatin regulatory protein that mainly functions by acetylating H4K16 [11–13]. KAT8 participates in cell proliferation, tumor invasion, DNA repair, autophagy and transcription regulation [13–18]. The effects of post-translational modification (PTM) of KAT8 on cellular processes and tumor progression have received increasing attention [19–22]. MOF-T392 phosphorylation mediated by ataxia telangiectasia-mutated (ATM) was reported to regulate 53BP1-mediated double-strand break repair pathway choice [19]. Mass spectrometry was used to map the lysines in KAT8, which are ubiquitylated by MSL2 in vitro and identified ubiquitylation sites of KAT8 in male and female cells of Drosophila melanogaster in vivo [21]. Also, KAT8 autoacetylation was shown in few studies [11, 13, 23]. For example, Lu Lu and his colleagues reported that KAT8 auto-acetylated at K274 residue in vitro and in vivo, and SIRT1 negatively modulates this process through regulating KAT8 recruitment to the chromatin [11]. However, little is known about the effects of KAT8 or KAT8 acetylation besides autoacetylation on lipolysis in colorectal cancer cells.

Lipolysis is a catabolic branch of the fatty acids (FA) cycle that provides FAs to meet metabolic need and removes them when in excess. Lipolysis hydrolyses the ester bonds of the triglyceride (TG) and plays a critical role in cell metabolism homoeostasis. Neutral hydrolysis of TGs to FAs and glycerol requires three consecutive steps and at least three different enzymes are involve, including adipose triglyceride lipase (ATGL), hormone-sensitive lipase (HSL) and monoacylglycerol lipase (MGL) [24–26]. ATGL catalyzes the initial step of lipolysis, catabolizing TGs to diacylglycerols (DGs). HSL, the main DG lipase, is rate-limiting for catabolism in adipose and non-adipose tissue and coordinates with ATGL to affect both the first two steps of TG breaking down, which mainly hydrolyze DGs to generate monoglycerides (MGs) and free FAs [27–29]. MGL is selectively responsible for the irreversible hydrolysis of monoglyceride (MGs) deriving from both extracellular and intracellular TG hydrolysis [30]. Lipolysis produces free fatty acid and then uses for β-oxidation and ATP production, which involves in various diseases such as cancer, type 2 diabetes and fatty liver disease [31].

Our previous study has reported that histone deacetylase SIRT6 plays a key role to regulate Cardiolipin *de novo* biosynthesis and fatty acid β-oxidation [9, 10]. Here, we are interested in the effect of histone acetyltransferase KAT8 on lipid metabolism. We identified KAT8 as an acetylated protein and KAT8 acetylation can be dynamically regulated by deacetylase SIRT6 and acetyltransferase GCN5, which is independent of its auto-acetylation. The K168/175 acetylation of KAT8 attenuates the binding activity of KAT8 and inhibits the recruitment of RNA pol II to the promoter region of the lipolysis-related genes *ATGL* and *HSL*, subsequently downregulating their expression to mediate lipolysis. Taken together, our data reveals that KAT8 acetylation plays a critical role in lipolysis to further affect invasive and migratory potential of CRC cells, and it may serve as a potential target for colorectal cancer therapy.

## Results

### KAT8 regulates lipolysis accompanied by enhanced KAT8 acetylation after PA treatment in human CRC cells

**Fig. 1:**
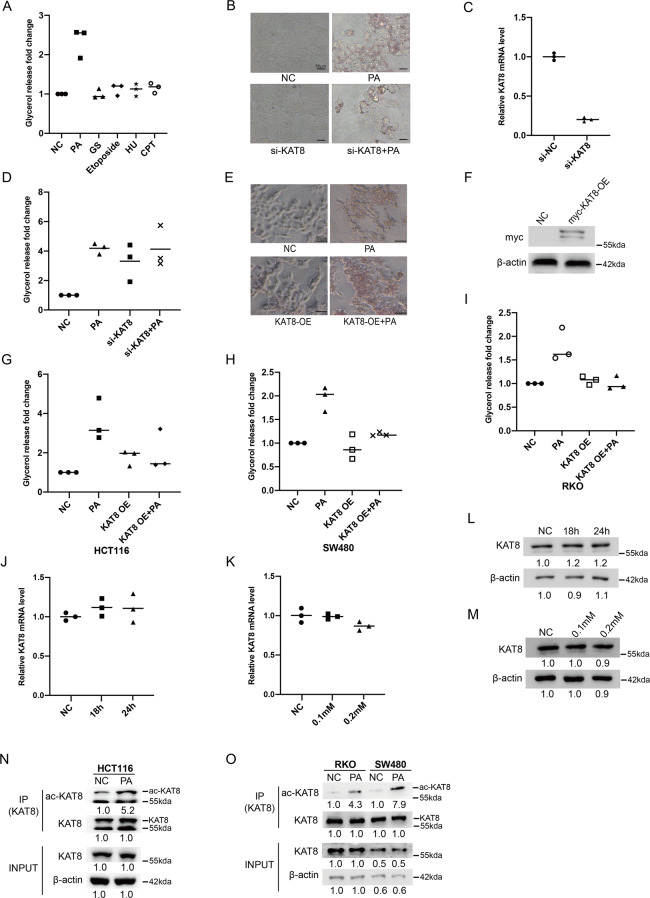
KAT8 regulates lipolysis accompanied by enhanced KAT8 acetylation after PA treatment in human CRC cells. **A** HCT116 cells were treated with PA (0.2 mM) for 24 h, glucose starvation (GS) for 18 h, etoposide (40 μM) for 8 h, HU (2 mM) for 2 h and CPT (1 μM) for 1 h. Cells were lysed and subjected to quantify the amount of glycerol inside cells by glycerol colorimetric assay kit. **B**, **E** HCT116 cells were transfected with KAT8 siRNA (B) or KAT8 plasmid (E), and then treated with or without PA at 0.2 mM for 24 h. Oil red O staining was used to detect the accumulation of lipid droplets. **C** The siRNA efficiency of KAT8 was detected by real-time PCR. **D**, **G** HCT116 cells were treated as outlined above, cells were lysed and subjected to quantify the amount of glycerol inside cells by glycerol colorimetric assay kit. **F** The transfected efficiency of KAT8 was detected by Western blotting. **H**, **I** SW480 (H) or RKO (I) cells were treated as outlined above, cells were lysed and subjected to quantify the amount of glycerol inside cells by glycerol colorimetric assay kit. **J**, **K** HCT116 cells were treated with 0.2 mM PA for 0, 18 h and 24 h (**J**) or PA (0.1 mM and 0.2 mM) for 24 h (**K**). The mRNA expression of KAT8 was analyzed by real-time PCR. mRNA levels of the control sample were set as 1, and relative mRNA levels of the experimental samples were normalized to this control. **L**, **M** HCT116 cells were stimulated under the same condition above. The total protein expression of KAT8 was detected by Western blotting. β-actin was used as a loading control. **N**, **O** HCT116 (N), SW480 and RKO (**O**) cells were treated with or without 0.2 mM PA for 24 h, cell lysates were then extracted for a Co-IP assay to detect endogenous acetylation levels of KAT8 in these cells. The bar (−) represents the means (*n* = 3).

KAT8 is essential for cell proliferation and plays a critical role in multiple physiological process [32, 33]. However, its role on lipolysis is still unclear. Several stimuli were used to detect their function on lipolysis in colon cancer HCT116 cells. As shown in Fig. 1A, only palmitic acid (PA) can effectively increase the release of glycerol showing that PA involves in lipolysis of colon cancer cells. Next, we explored the relationship between KAT8 and lipolysis of CRC cells during PA stimulation. The lipid accumulation significantly reduced in KAT8 siRNA cells compared with control cells after PA treatment (Fig. 1B, C). The glycerol release experiments also showed that KAT8 may play a role in lipolysis induced by PA stimulation in HCT116 cells (Fig. 1D). In the meanwhile, opposite results were shown in PA-treated KAT8 overexpression cells, indicating that KAT8 overexpression inhibits lipolysis by PA stimulation (Fig. 1E–G). Also, the key regulation effect of KAT8 in lipolysis was found in colon cancer RKO and sw480 cells (Fig. 1H, I).

Given that KAT8 involves in the process of lipolysis, we next detected the mRNA and protein levels of KAT8 after PA treatment. No significant difference was found in both KAT8 mRNA and protein levels after PA treatment (Fig. 1J–M). Remarkably, the endogenous acetylation of KAT8 increased sharply after PA treatment in these CRC cells, suggesting that KAT8 acetylation might be play a critical role in regulating lipolysis in CRC cells (Fig. 1N, O). Meanwhile, the familiar phenomena were shown in other cancer cells, such as hepatocellular carcinoma HepG2 cells, gastric carcinoma MGC-803 cells and pancreatic cancer PANC1 cells (Supplementary Fig. S1A, 1C–E). However, KAT8 acetylation was shown not significantly increased in human embryonic lung diploid fibroblasts 2BS cells (Supplementary Fig. S1B). These data suggest that KAT8 acetylation involved in the process of lipolysis is universal in cancer cells.

### GCN5 is an acetyltransferase of KAT8

**Fig. 2:**
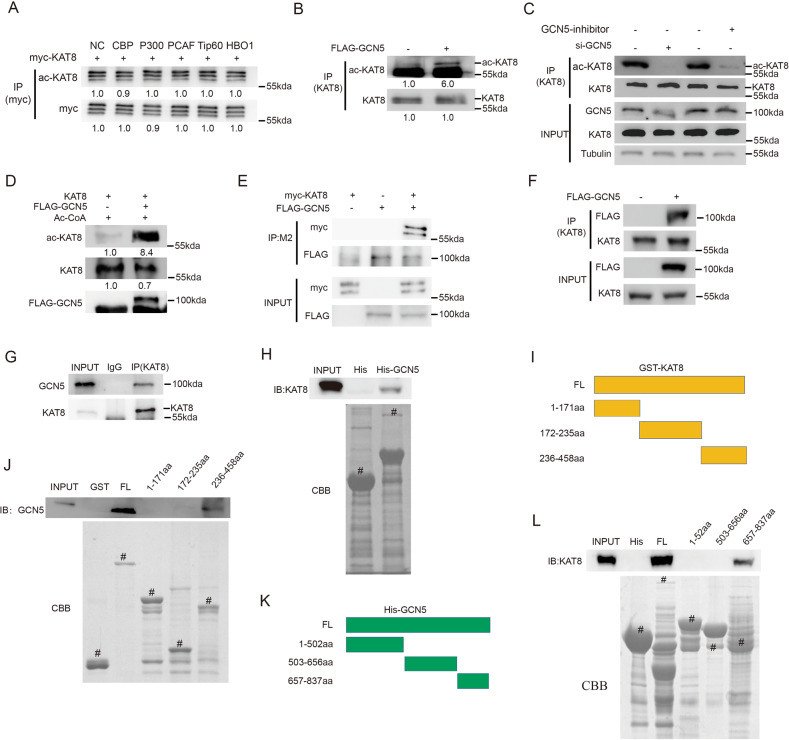
GCN5 is an acetyltransferase of KAT8. **A**, **B** KAT8 was co-transfected with several critical acetyltransferases in HCT116 cells and then Co-IP was performed to detect the level of KAT8 acetylation. **C** HCT116 cells were transfected with GCN5 siRNA or treated with GCN5 inhibitor MB-3, and then Co-IP was performed to detect the level of KAT8 acetylation. **D** Flag-GCN5 was transfected into HCT116 cells and then KAT8 and Flag-GCN5 proteins were purified to perform in vitro acetylation assay. The level of KAT8 acetylation was detected by Western blotting. **E** HCT116 cells were co-transfected with Flag-GCN5 and myc-KAT8 and then proteins were extracted for Co-IP to detect the exogenous interaction between KAT8 and GCN5. **F** HCT116 cells were transfected with Flag-GCN5 and then proteins were extracted for Co-IP to detect the semi-exogenous interaction between KAT8 and GCN5. **G** Protein of HCT116 cells was extracted for Co-IP to detect the endogenous interaction between KAT8 and GCN5. **H** His-GCN5, GST-KAT8 and GST were expressed, and purified in bacteria. GST-pull down assays were performed to show the direct interaction between GCN5 and KAT8 in vitro. **I** Schematic of plasmids encoding KAT8. **J** GST-KAT8 FL or fragments were incubated with His-GCN5, and Western blotting or Coomassie staining was performed to detect the direct binding of KAT8 and GCN5 in vitro. **K** Schematic of plasmids encoding GCN5. **L** His-GCN5 FL or fragments were incubated with GST-KAT8, and Western blotting or Coomassie staining was performed to detect the direct binding of GCN5 and KAT8 in vitro. ^#^ indicates the specific bands.

Since KAT8 acetylation might play a critical role in regulating lipolysis in CRC cells, we next aimed to identify the specific acetyltransferase by screening a series of acetyltransferases. We co-transfected KAT8 with different histone acetyltransferases (HATs) and found that only GCN5 dramatically increased the acetylation level of KAT8 (Fig. 2A, B). KAT8 acetylation was also dramatically decreased when the cells were treated with GCN5 siRNA or GCN5 enzymatic inhibitor MB-3 (Fig. 2C). In vitro acetylation assay further confirmed that GCN5 is the acetyltransferase of KAT8 (Fig. 2D).

Next, we detected whether there is a molecular link between GCN5 and KAT8. Exogenous, semi-exogenous and endogenous co-immunoprecipitation showed the interaction of GCN5 with KAT8 (Fig. 2E–G). We then performed a GST pull-down assay to investigate whether KAT8 directly interacts with GCN5. We found that GCN5 directly interacts with GST-KAT8 but not GST alone, showing that GCN5 can interact with KAT8 in vitro (Fig. 2H). In the process of mapping the regions of KAT8 involves in GCN5 binding, we found that the N-terminus domain of KAT8 is responsible for the interaction with GCN5 (Fig. 2I, J). Also, N-terminus fragment of GCN5 was confirmed to be responsible for this interaction (Fig. 2K, L). Collectively, these experiments indicate that GCN5 can interact with KAT8 and acetylate KAT8 in vivo and in vitro.

### SIRT6 is a deacetylase of KAT8 and is responsible for KAT8 acetylation

**Fig. 3:**
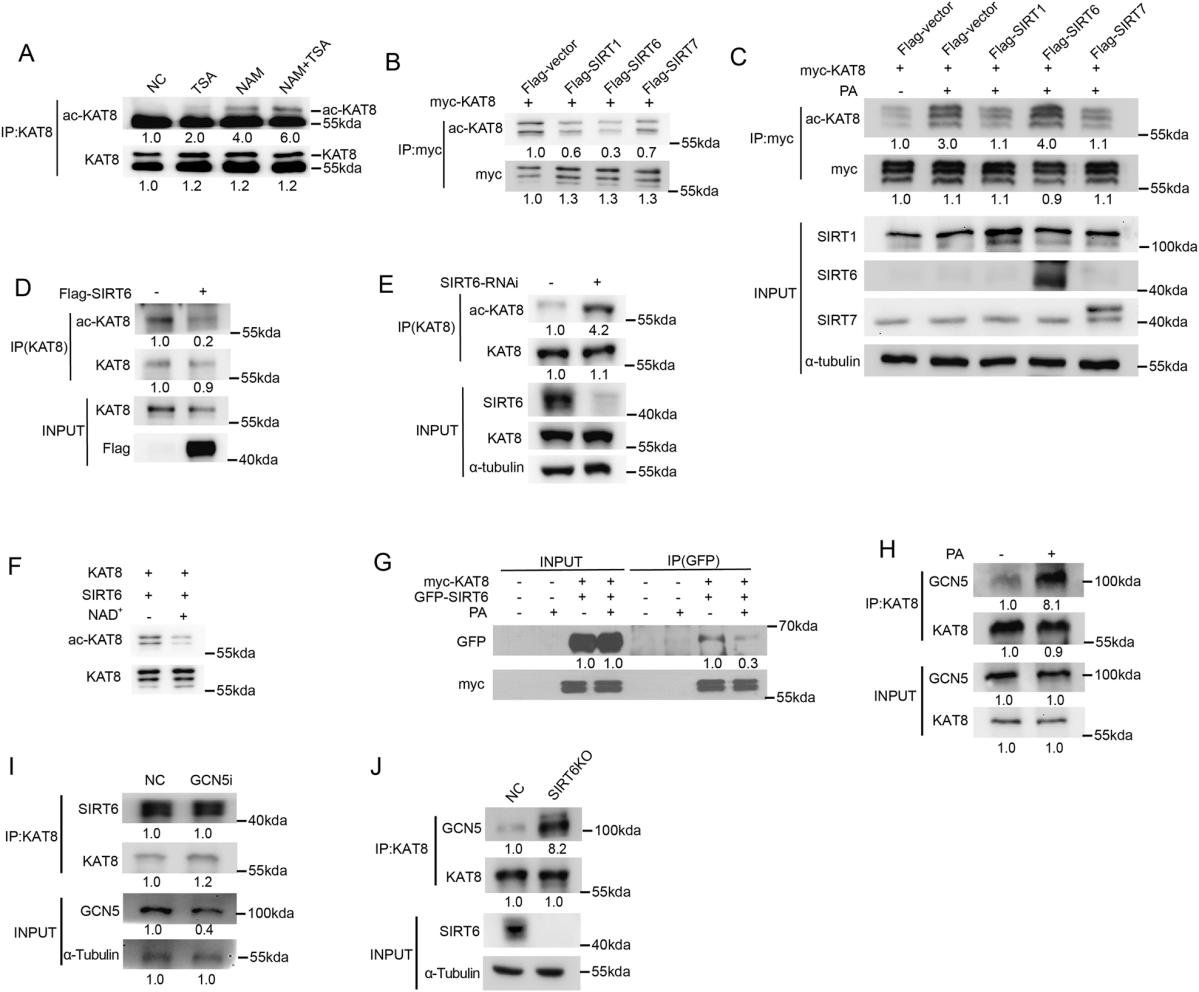
SIRT6 is a deacetylase of KAT8 and is responsible for KAT8 acetylation. **A** HCT116 cells were treated with 1 μM TSA or 5 mM NAM for 8 h and then proteins were extracted for Co-IP assay to detect the level of KAT8 acetylation. **B** HCT116 cells were co-transfected with myc-KAT8, Flag-SIRT1, Flag-SIRT6 or Flag-SIRT7, and then proteins were extracted for Co-IP to detect the level of KAT8 acetylation. **C** HCT116 cells were co-transfected with the plasmids above, and then treated with PA at 0.2 mM for 24 h. Proteins were extracted for Co-IP to detect the level of KAT8 acetylation. **D**, **E** HCT116 cells were transfected with Flag-SIRT6 (**D**) or SIRT6 siRNA (**E)**, and proteins were extracted for Co-IP to detect the level of KAT8 acetylation. **F** myc-KAT8 and Flag-SIRT6 were co-transfected into HCT116 cells, and then KAT8 and SIRT6 proteins were purified to perform in vitro deacetylation assay. **G**, **H** HCT116 cells were co-transfected with GFP-SIRT6 (**G**) or Flag-GCN5 (**H**) and myc-KAT8, and then treated with PA at 0.2 mM for 24 h. Cell lysates were extracted for Co-IP assay to detect the exogenous interaction between SIRT6 (**G**) /GCN5 (**H**) and KAT8. **I** HCT116 was transfected with GCN5 siRNA or non-specific RNA as control, protein was then extracted for Co-IP to detect the endogenous interaction between KAT8 and SIRT6. **J** HCT116 SIRT6-KO cells were extracted for Co-IP to detect the endogenous interaction between KAT8 and GCN5.

We next sought to identify the deacetylase of KAT8 acetylation. HDACI, II family inhibitor TSA and the Sirtuin family inhibitor nicotinamide were used and we found that nicotinamide significantly increased the acetylation level of KAT8, suggesting that Sirtuins might be the major deacetylase of KAT8 (Fig. 3A). Subcellular location of KAT8 showed that KAT8 is mainly distributed in nucleus (data not shown). Thus, we co-transfected myc-tagged KAT8 together with Flag-tagged SIRT1/6/7 into HCT116 cells. As shown in Fig. 3B, the deacetylase activity of SIRT1 or SIRT6 on KAT8 acetylation was stronger than SIRT7 in transfected HCT116 cells. Interestingly, KAT8 acetylation was only recovered in SIRT6 transfected HCT116 cells after PA treatment, implying that SIRT6 may have certain relationship with KAT8 acetylation after PA treatment (Fig. 3C). When SIRT6 siRNA or Flag-SIRT6 plasmid was transfected into HCT116 cells, KAT8 acetylation was changed sharply compared with control group (Fig. 3D, E). Also, the data of in vitro deacetylation assay suggested that SIRT6 significantly deacetylates KAT8 in vitro (Fig. 3F). These data indicated that SIRT6 is the deacetylase of KAT8.

To clarify which is the key mediator of KAT8 acetylation, we firstly monitored the interaction between GCN5/SIRT6 and KAT8 after PA treatment. The interaction between SIRT6 and KAT8 was sharply reduced accompanied by the increased interaction between GCN5 and KAT8 after PA treatment, showing that KAT8 acetylation might be dynamically regulated by the balance of GCN5 and SIRT6 (Fig. 3G, H). Remarkably, the interaction between SIRT6 and KAT8 did not change in GCN5-specific siRNA-treated cells (Fig. 3I). On the contrary, the interaction between GCN5 and KAT8 increased significantly in SIRT6 KO cells showing that SIRT6 is the key mediator on KAT8 acetylation after PA treatment (Fig. 3J).

### KAT8 interacts with SIRT6 in vivo and in vitro

**Fig. 4:**
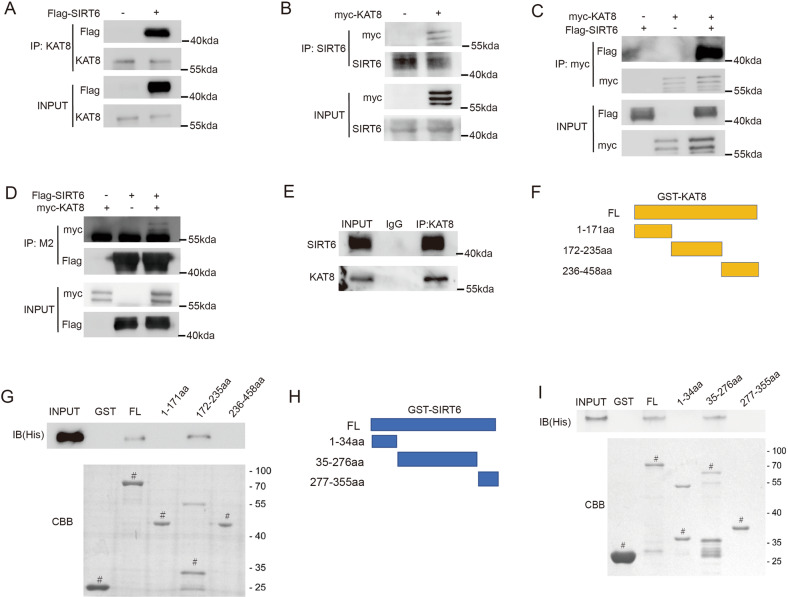
KAT8 interacts with SIRT6 in vivo and in vitro. **A**, **B** HCT116 cells were transfected with Flag-SIRT6 plasmids (A) or myc-KAT8 plasmids (B), and then proteins were extracted for Co-IP to detect the semi-exogenous interaction between KAT8 and SIRT6. **C**, **D** HCT116 cells were transfected with Flag-SIRT6 and/or myc-KAT8 plasmids, and then proteins were extracted for Co-IP to detect the exogenous interaction between KAT8 and SIRT6. **E** Protein of HCT116 cells was extracted for Co-IP to detect the endogenous interaction between KAT8 and SIRT6. **F** Schematic of plasmids encoding KAT8. **G** GST-KAT8 FL or fragments were incubated with His-SIRT6, and Western blotting or Coomassie staining was performed to detect the direct binding of KAT8 and SIRT6 in vitro. **H** Schematic of plasmids encoding SIRT6. **I** GST-SIRT6 FL or fragments were incubated with His-KAT8, and Western blotting or Coomassie staining was performed to detect the interaction. ^#^ indicates the specific bands.

Having established that SIRT6 is a major deacetylase of KAT8, we aimed to clarify the molecular link between SIRT6 and KAT8. Exogenous, semi-exogenous and endogenous co-immunoprecipitation presented the interaction of SIRT6 with KAT8 (Fig. 4A–E). GST pull-down assay was performed to investigate whether the interaction between KAT8 and SIRT6 is direct. We found that KAT8 core domain is responsible for this interaction (Fig. 4F, G). SIRT6 CD domain was also confirmed to be responsible for the interaction (Fig. 4H, I). Together, these results indicate that KAT8 directly interacts with SIRT6 in vivo and in vitro.

### Lysine 168 and lysine 175 are the major acetylation sites of KAT8 and mediate lipolysis of CRC cells

**Fig. 5:**
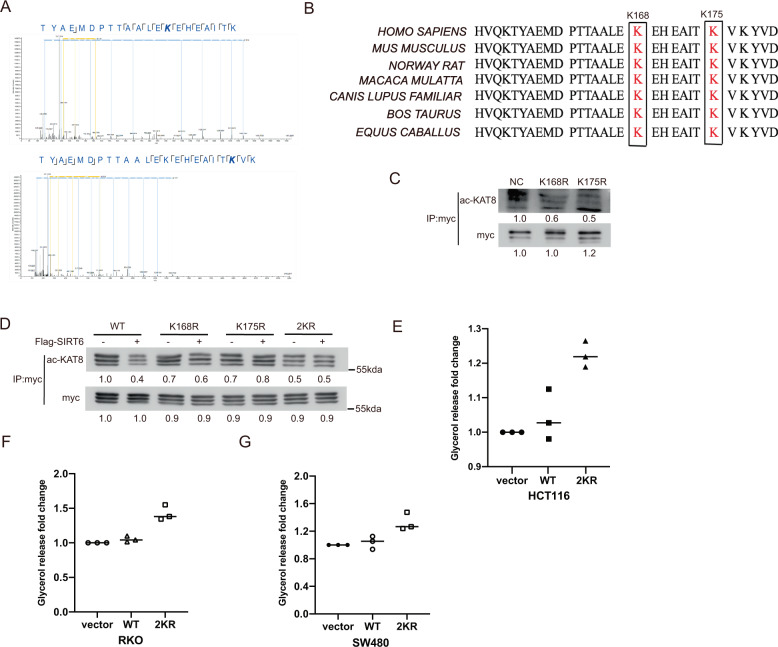
Lysine 168 and lysine 175 are the major acetylation sites of KAT8 and mediate lipolysis of CRC cells. **A** HCT116 cells were transfected with myc-KAT8 and then treated with PA at 0.2 mM for 24 h. Myc-KAT8 was purified and subsequently separated by SDS-PAGE and stained with CBB. The KAT8 band was analyzed by mass spectrometry. **B** Alignment of MS-characterized putative KAT8 acetylation residues among different species. **C** HCT116 cells were transfected with myc-KAT8 WT, K168R or K175R mutant plasmids for a Co-IP assay to detect the level of KAT8 acetylation. **D** HCT116 cells were transfected with the plasmids above combined with Flag-SIRT6 for a Co-IP assay to detect the level of KAT8 acetylation. **E-G** HCT116 (**E**), RKO (**F**) and SW480 (**G**) cells were transfected with myc-vector, myc-KAT8 WT or myc-KAT8 2KR mutant plasmids, and then cells were lysed to quantify the amount of glycerol inside cells by glycerol colorimetric assay kit. The bar (−) represents the means (*n* = 3).

Next, we identified the major acetylation sites of KAT8 by mass spectrometry (MS). The MS data indicated that KAT8 is acetylated at lysine 168 and lysine 175 residues after PA treatment (Fig. 5A). These two sites are highly conserved from *EQUUS* to humans (Fig. 5B). To further confirm the acetylation sites of KAT8, lysine to alanine mutant KAT8-K168R, KAT8-K175R or KAT8-2KR plasmids were generated and transfected into HCT116 cells. As shown in Fig. 5C, KAT8 acetylation was reduced significantly after PA stimulation in both mutated KAT8 transfected cells. Also, the results of co-transfected with Flag SIRT6 plasmids showed that the reduction of KAT8 acetylation in KAT8 WT transfected cells was completely recovered in KAT8 KR transfected cells (Fig. 5D). At the same time, the glycerol releases in KAT8-2KR overexpression HCT116, RKO and SW480 cells were significantly increased comparing with KAT8 WT transfected cells (Fig. 5E–G). These data demonstrate that K168 and K175 are two major functional sites of KAT8 acetylation and involve in mediating lipolysis of CRC cells.

### KAT8 regulates the expression of lipolysis-related genes *via* its acetylation

**Fig. 6:**
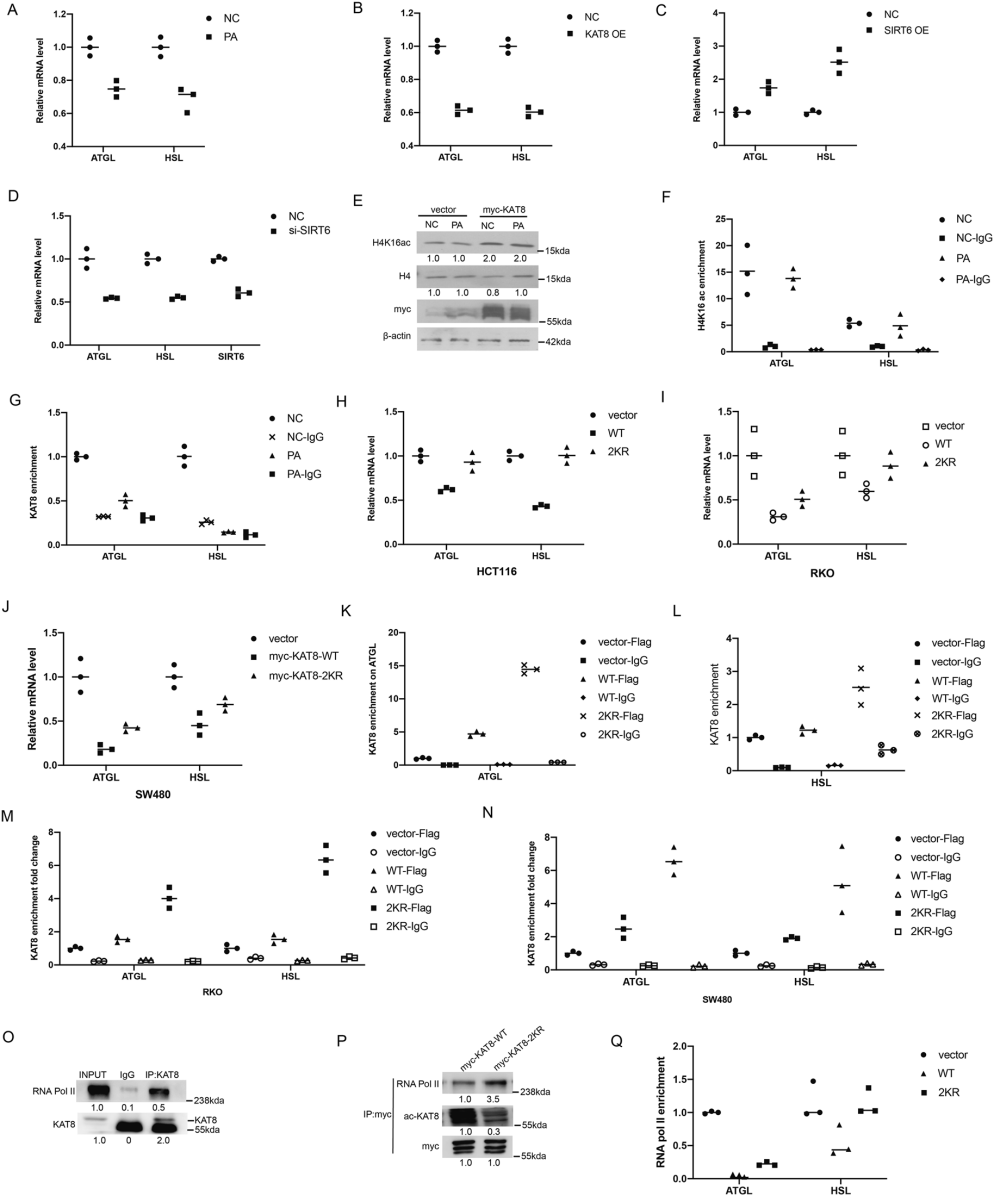
KAT8 regulates the expression of lipolysis-related genes via its acetylation. **A** HCT116 cells were treated with PA at 0.2 mM for 24 h, and then the expression of *ATGL* and *HSL* were analyzed by real-time PCR. **B** HCT116 cells were transfected with myc-KAT8 to detect the expression of *ATGL* and *HSL* by real-time PCR. **C**, **D** HCT116 cells were transfected with Flag-SIRT6 (C) or SIRT6 siRNA (D) for 48 h, and then the expression of *ATGL* and *HSL* were analyzed by real-time qPCR. **E** HCT116 cells were transfected with pCMV-myc-vector or myc-KAT8 and then treated with PA to determine the changes in H4K16 acetylation patterns by Western blotting. H4 is shown as a loading control. **F**, **G** HCT116 cells were treated with or without PA at 0.2 mM for 24 h. ChIP assay was performed to detect the enrichment of H4K16ac (F) and KAT8 (G) at the gene promoter regions of *ATGL* and *HSL*. **H-J** HCT116 (H), RKO (I) and SW480 (J) cells were transfected with myc-KAT8 WT or myc-KAT8 2KR mutant plasmid for 48 h. The expression of *ATGL* and *HSL* was analyzed by real-time PCR. **K-N** HCT116 (K, L), RKO (M) and SW480 (N) cells were transfected with the Flag-vector, Flag-KAT8-WT, Flag-KAT8-2KR plasmids, ChIP assay was performed to detect the enrichment of KAT8 on the gene promoter regions of *ATGL* and *HSL*. **O** Protein of HCT116 cells was extracted for Co-IP to detect the endogenous interaction between KAT8 and RNA pol II. **P** HCT116 cells were transfected with myc-KAT8 WT or myc-KAT8 2KR mutant plasmid for Co-IP to detect the interaction between KAT8 and RNA pol II. **Q** HCT116 cells were transfected with the plasmids mentioned above, ChIP assay was performed to detect the enrichment of RNA pol II at the gene promoter regions of *ATGL* and *HSL*. mRNA levels of the control sample were set as 1, and relative mRNA levels of the other samples were normalized to this control. The bar (−) represents the means (*n* = 3).

Having known that KAT8 acetylation has the ability to regulate lipolysis of CRC cells, we next explored the mechanism under this process. ATGL and HSL are two key enzymes responsible for lipolysis. We firstly detected the expression of *ATGL* and *HSL* genes and found that both genes reduced after PA stimulation (Fig. 6A). Also, the key role of SIRT6 or KAT8 was shown in regulating the expression of *ATGL* and *HSL* genes (Fig. 6B–D).

Next, we try to elucidate the mechanism how KAT8 regulates the expression of lipolysis-related genes after PA treatment. Histone H4K16ac is a specific acetylation target of KAT8, so we firstly detected the expression of H4K16ac after PA treatment. No significant difference was detected in global H4K16ac level or in the enrichment of H4K16ac to the promoter of *ATGL* and *HSL* genes, suggesting that KAT8 may not decrease the expression of *ATGL* and *HSL* genes through H4K16ac (Fig. 6E, F). Interestingly, the binding affinity of KAT8 to the promoter of *ATGL* and *HSL* genes was found reduced sharply after PA treatment, demonstrating that KAT8 regulates the expression of both genes by changing its recruitment to the gene promoters (Fig. 6G). We then confirmed the effect of KAT8 acetylation on gene expression of CRC cells. The expression of *ATGL* and *HSL* were reduced in wild-type KAT8-transfected cells and recovered in 2KR mutant KAT8-transfected cells (Fig. 6H–J). ChIP qPCR assay revealed that the binding activity of KAT8 to the promoters of both *ATGL* and *HSL* genes were significantly increased in the 2KR mutant KAT8-transfected CRC cells comparing with control group (Fig. 6K–N). These data indicate that KAT8 acetylation mediates the binding activity of KAT8 to the promoter of lipolysis-related genes to regulate their expression in CRC cells.

To further explore the mechanism of KAT8 acetylation on regulating *ATGL* and *HSL* gene expression, we screened the possible regulatory factors, and identified that KAT8 can interact with RNA polymerase II (RNAP II) (Fig. 6O). Remarkably, the interaction between KAT8 and RNAP II was increased in the 2KR mutant KAT8-transfected cells comparing with control cells (Fig. 6P). Also, we found that the reduction of binding activity of RNAP II on the promoters of *ATGL* and *HSL* in wild-type KAT8-transfected cells was recovered in 2KR mutant KAT8-transfected cells (Fig. 6Q). Collectively, these experiments indicate that KAT8 may serve as a co-transcription factor of RNAP II to regulate the expression of lipolysis-related genes and KAT8 acetylation is response for this process through mediating KAT8 binding activity to the promoters of these genes.

### KAT8 acetylation regulates migration and invasion of CRC cells

**Fig. 7:**
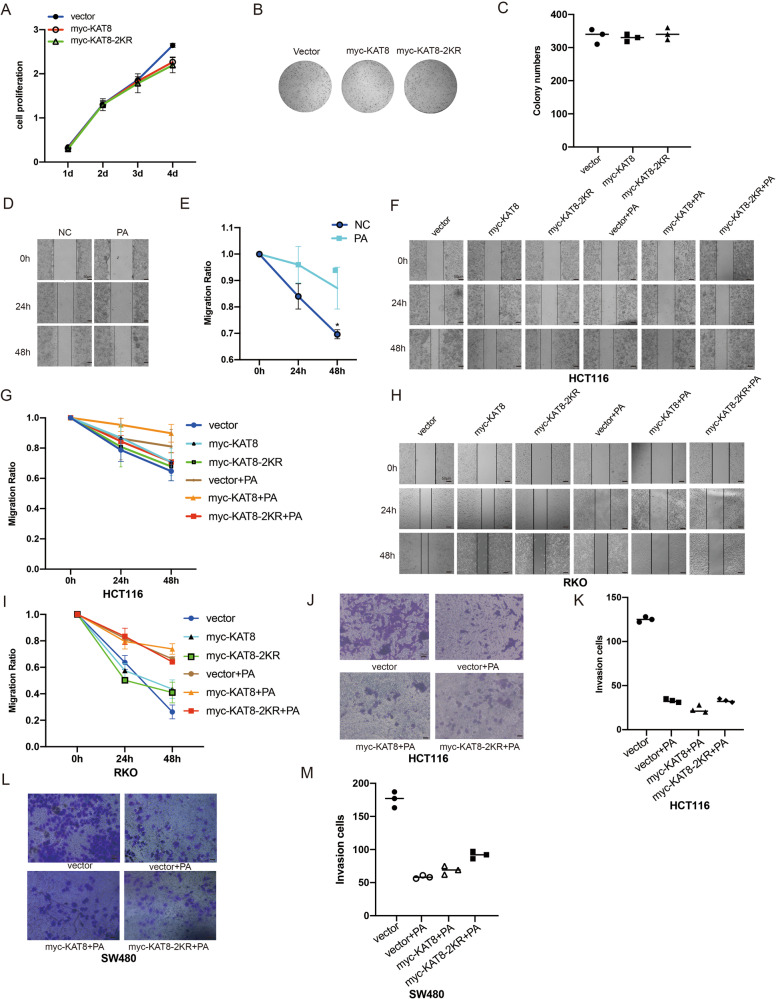
KAT8 acetylation regulates migration and invasion of CRC cells. **A** HCT116 cells were transfected with pCMV-myc-vector, myc-KAT8-WT and myc-KAT8-2KR plasmids for CCK8 assay to generate a growth curve. **B**, **C** HCT116 cells were transfected with the plasmids mentioned above, colony formation assay was performed to identify visible colonies. **D**, **E** HCT116 cells were treated with PA at 0.2 mM for 24 h and 48 h. A wound-healing assay was performed to evaluate the migration ability of cells. **F-I** HCT116 (F, G) and RKO (H, I) cells were transfected with the plasmids mentioned above, and treated with or without PA at 0.2 mM for 24 h. A wound-healing assay was performed to evaluate the migration ability of cells. **J-M** HCT116 (J, K) and RKO cells (L, M) were transfected with the plasmids mentioned above, a transwell assay was performed to evaluate the invasive ability of cells. The bar (−) represents the means (*n* = 3).

We further demonstrated the functional significance of KAT8 acetylation in CRC progression. The results of CCK-8 viability assay and colony formation assay showed that KAT8 acetylation is not affected the proliferation of CRC cells (Fig. 7A–C). Next, a wound-healing assay was performed and we found that the rate of closure was significantly reduced after PA treatment (Fig. 7D, E). Remarkably, a significantly reduced rate of closure was found in the KAT8-WT cells compared with that in the KAT8-2KR cells after PA stimulation, indicating that KAT8 acetylation plays an important role on PA-stimulated HCT116 cell migration (Fig. 7F, G). Similar results were produced when using RKO cells (Fig. 7H, I). Also, a matrigel-coated invasion assay was established and the degree of cellular invasion was much higher in KAT8-2KR cells than KAT8-WT transfected HCT116 cells after PA stimulation (Fig. 7J, K). Similar results were produced when using SW480 cells (Fig. 7L, M). Taken together, these results suggest that KAT8 acetylation can effectively inhibit migration and invasion of CRC cells.

### KAT8 acetylation regulates migration and invasion through Lipase HSL in CRC cells

**Fig. 8:**
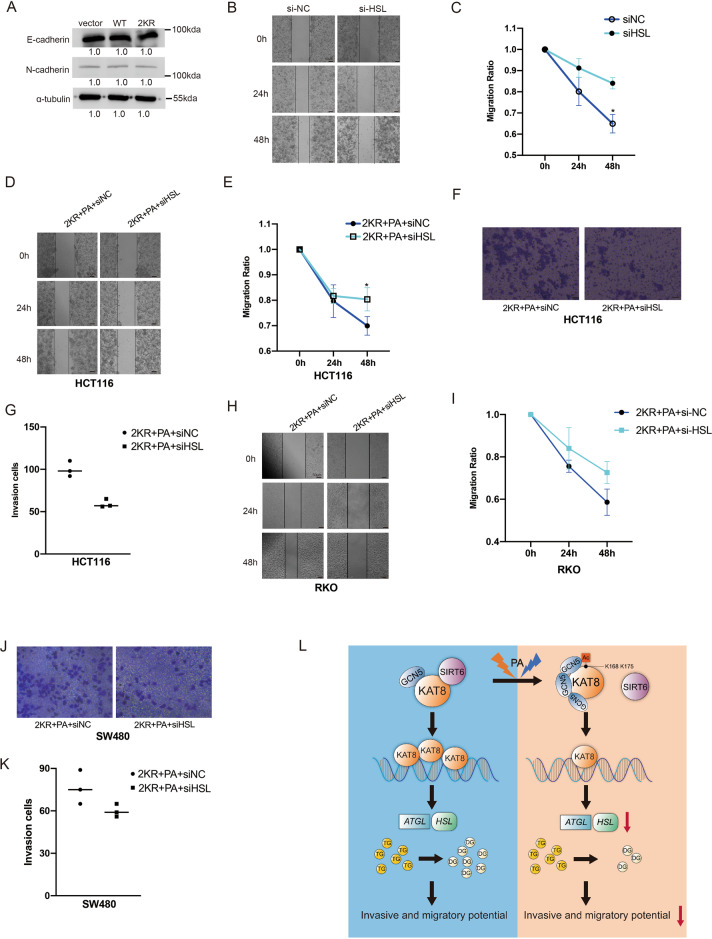
KAT8 acetylation regulates migration and invasion through lipolysis in CRC cells. **A** HCT116 cells were transfected with pCMV-myc-vector, myc-KAT8-WT and myc-KAT8-2KR plasmids, and Western blotting was performed to detect the expression of the EMT marker E-cadherin and N-cadherin. **B**, **C** HCT116 cells were transfected with HSL siRNA or non-specific siRNA for 48 h. A wound-healing assay was performed to evaluate the migration ability of cells. **D**, **E** HCT116 cells were transfected by myc-KAT8-2KR firstly and treated with PA at 0.1 mM for 24 h after HSL siRNA or non-specific siRNA were transfected into HCT116 cells. A wound-healing assay was performed to evaluate the migration ability of each group. **F**, **G** HCT116 cells were treated as mentioned above, a transwell assay was performed to evaluate the invasive ability of each group. **H**, **I** RKO cells were transfected by myc-KAT8-2KR firstly and then treated with PA at 0.1 mM for 24 h after HSL siRNA or non-specific siRNA were transfected into RKO cells. A wound-healing assay was performed to evaluate the migration ability of each group. **J**, **K** SW480 cells were treated as mentioned above, a transwell assay was performed to evaluate the invasive ability of each group. **L** A schematic showing a possible mechanism that KAT8 acetylation-controlled lipolysis affects the invasive and migratory potential of colorectal cancer cells.

Finally, we explored the mechanism insight into KAT8 acetylation-dependent cell migration and invasion. The expression of E-cadherin and N-cadherin, the main marker of epithelial-mesenchymal transition (EMT), were firstly examined and no significant changes were found in both KAT8-WT and KAT8-2KR transfected cells, implying that KAT8 acetylation-dependent cell migration and invasion is not driven by an EMT process (Fig. 8A). Lipase HSL was reported to regulate the invasiveness of pancreatic cancer cells [34], so we examined the effect of HSL on CRC cells migration and invasive. As shown in Fig. 8B, C, knock down HSL was found to effectively inhibit the migration of HCT116 cells. Also, HCT116 cells were transfected with myc-KAT8-2KR firstly and treated with PA at 0.1 mM for 24 h after HSL siRNA or non-specific siRNA were transfected into these cells. A wound-healing assay and matrigel-coated invasion assay were performed to evaluate the migration and invasion ability of HSL in HCT116 cells. These data showed that knocking down HSL effectively inhibited KAT8 (2KR) promoted the migration and invasion of HCT116 cells by PA stimulation (Fig. 8D–G). Also, the familiar phenomena were shown in RKO and SW480 cells (Fig. 8H–K). Together, these data demonstrate that KAT8 acetylation regulates migration and invasion through HSL in CRC cells.

## Discussion

KAT8 is associated with a wide range of cellular functions [35, 36]. Here, we found that PA treatment stimulates the release of glycerol and overexpression of KAT8 inhibits this process, showing that KAT8 involves in lipolysis of CRC cells (Fig. 1). To further mechanistically study, we found that KAT8 acetylation is response for regulating the recruitment of RNA polymerase II to the *ATGL* and *HSL* promoters to decrease the expression of these genes and further affect invasive and migratory potential in CRC cells (Fig. 8L). Recently, the effect of KAT8 on lipid metabolism has been paid on more and more attention. KAT8 was shown to acetylate fatty acid synthase (FASN) to further destabilize FASN and decrease de novo lipogenesis and tumor cell growth of human hepatocellular carcinoma [37]. Gao et al. found that signal transducer and activator of transcription 5B (STAT5B) can modulate adipocyte differentiation *via* KAT8 [38]. Also, KAT8 was reported to active fatty acid oxidation (FAO) to block acquisition of quiescence in ground-state ESCs [39]. Our data show the new function of KAT8 on lipid catabolism, and indicate the important role of KAT8 acetylation in human CRC progression.

In the process of identifying the acetyltransferases and deacetylase of KAT8, we found that GCN5 is main acetyltransferases of KAT8 (Fig. 2). Also, SIRT6 is confirmed as a deacetylase of KAT8 by PA stimulation (Fig. 3C–F). It is interesting to know which is the key mediator of KAT8 acetylation. We speculate that there is a dynamic balance between SIRT6 and GCN5 on KAT8 acetylation. GCN5 and SIRT6 can both interact with KAT8 in physiological condition. When SIRT6 senses the stress of lipid metabolism after PA treatment, the interaction between SIRT6 and KAT8 was significantly reduced. Meanwhile, the interaction between GCN5 and KAT8 was magically increased resulting in the increasing of KAT8 acetylation (Fig. 3G–J), indicating that SIRT6 is the key mediator on KAT8 acetylation. In our study, the acetylated sites of KAT8 are K168 and K175 residues after PA treatment. We brought up the hypothesis that auto-acetylation of KAT8 might not happened on these two residues, so SIRT6 and GCN5 are needed for the acetylation of KAT8. Here, we reported for the first time that KAT8 acetylation can be dynamically regulated by SIRT6 and GCN5 instead of its auto-acetylation after PA treatment. It adds a new layer of knowledge on KAT8 acetylation and it will be beneficial for us to further understand the molecular role of KAT8.

KAT8 was identified as a histone H4K16-specific acetyltransferase [15, 40, 41]. Knock-down KAT8 resulted in silencing of the expression of target of methylation-mediated silencing (*TMS1*) gene, showing that KAT8-dependent histone H4K16ac was important in the maintenance of TMS1 gene activity [42]. However, genome-wide H4K16ac distribution was analyzed and identified 25,893 DNA regions in HEK293 cells by ChIP assay indicating that centromeric regions of chromosome are largely free of H4K16ac and only a small fraction (~10%) is found near promoters [43]. The results of this study imply that KAT8 mediated H4K16ac may be secondary on transcription regulation. In our study, we found that KAT8 decreased the expression of *ATGL* and *HSL* genes was not through H4K16ac (Fig. 6E, F). Remarkably, the binding activity of KAT8 on the promoter of both genes was sharply reduced after PA treatment (Fig. 6G). Importantly, KAT8 acetylation was shown to involve in this process and played an important role in regulating the expression of lipolysis-related genes (Fig. 6H–Q). These experiments provide an evidence that KAT8 may serve as a transcription co-activator to regulate the expression of lipolysis-related genes. More experiments are needed to further confirm the effect of KAT8 as a transcription co-factor on gene expression.

KAT8 depletion was shown to promote migration and invasion of tumor cells recently [44]. Here, we assessed the effect of KAT8 acetylation on CRC progression. The results showed that KAT8 acetylation effectively inhibited PA-stimulated migration and invasion without affecting the proliferation of CRC cells (Fig. 7). To explore the mechanism insight into KAT8 acetylation-dependent cell migration and invasion, we found that knowing down HSL effectively inhibited KAT8 (2KR) promoted migration and invasion of CRC cells (Fig. 8D–K). The results of q-PCR and ChIP q-PCR assay showed that KAT8 acetylation attenuates the binding activity of KAT8 to the promoter of lipolysis-related gene *HSL* to further lead to the decreased expression of *HSL* gene and inhibit the migration and invasion of CRC cells (Fig. 6G–N). The data indicate that KAT8 acetylation might be regulate migration and invasion through HSL in CRC cells.

In conclusion, our data reports that KAT8 acetylation at K168/175 residues was dynamically regulated by SIRT6 and GCN5 during PA treatment. KAT8 suppresses *HSL* expression to further affect the invasive and migratory potential in colorectal cancer cells. Our data reveal a novel mechanism by which KAT8 regulates lipolysis and control metastatic invasion in CRC cells. These findings expand the field of protein epigenetic regulation on lipid metabolism to mediate metastasis of CRC cells and provide a potential target for colorectal cancer therapy.

## Materials and methods

### Cell lines, cell culture and reagents

Human colon cancer HCT116 and SW480 cells, human liver cancer HepG2 cells, human pancreas cancer PANC1 cells, human gastric carcinoma MGC803 cells, human embryonic lung diploid fibroblasts 2BS were cultured by Dulbecco Modified Eagle Medium (DMEM, Macgene, China) supplemented with 10% heat-inactivated fetal bovine serum (Gemini, south America). Human colon cancer RKO cells was cultured by RPMI-1640 supplemented with 5% FBS. All the cells were cultured with penicillin/Streptomycin in a 37 °C incubator with a humidified, 5% CO_2_ atmosphere. No signs of mycoplasma contamination were found for all cell lines. Short tandem repeat profiling was used for cell line authentication. Palmitic acid (PA) was purchased from SIGMA (St Louis, MO, USA) and then prepared to a stock solution and stored in room temperature.

### Plasmid construction and transfection

All the genes open reading frame were amplified from a cDNA library of HCT116 cells by PCR (R045A, TaKaRa, Japan) and cloned into 3x Flag CMV10, pCMV-myc, TF-pcold-His. The mutations of these genes were generated using a site-directed mutagenesis kit (FM111-01, transgene, China). Plasmids were transfected into cell lines by Hieff TransTM Liposomal Transfection Reagent (40802ES02, YEASEN, China) according to the manufacturer’s protocol.

### Oil red O staining

HCT116 cells were treated as required and staining with the Oil Red O working solution for 5 min. The stained lipid droplets were monitored under a microscope (Olympus, Tokyo, Japan).

### Glycerol colorimetric assay

HCT116, SW480, RKO, HepG2, MGC803 and PANC1 cells were treated as required and then lysed with the glycerol colorimetric assay kit (Applygen Technologies, Beijing, China). The lysates were then heated to 70 °C for 10 min to inactivate residue lipase activity. Glycerol in the lysates was determined by the enzyme-coupled GPO-Trinder reaction from the absorption of 550 nm.

### RNA extraction and RT-qPCR

Total RNA was extracted by Trizol agent (Applygen, China). cDNA was synthesized using Quantscript RT Kit (Promega,WI, USA) according to the instruction. RT-qPCR assay was performed by 7500 Fast RT-PCR machine with SYBR PCR mix agent (vazyme, China). The primers sequences used for RT-qPCR are available upon request.

### RNA interference (RNAi)

RNA interference was performed as described [45]. Cells were harvested after transfected with RNAi oligonucleotides and non-specific siRNA and subjected to Western blotting, RT-qPCR or a ChIP assay, respectively. The RNAi oligonucleotides sequences used are available upon request.

### Protein extraction and western blotting

Different cells were harvested after treatment and proteins were extracted to detect the expression by Western blotting as previously described with minor modifications [46]. Equal amounts of proteins were size fractionated by 9 to 15% sodium dodecyl sulfate (SDS)-polyacrylamide gel electrophoresis. Anti-SIRT6 (2590 S, Cell Signaling Techology, Danvers, MA, USA), anti-KAT8 (ab200660, abcam, Cambridge, MA, USA), anti-Acetyl-lysine (PTM-105RM, PTMBIO, China), anti-Flag (F1804, Sigma Aldrich), anti-SIRT1 (8469 S, Cell Signaling Techology, Danvers, MA, USA), anti-SIRT7 (5360 S, Cell Signaling Techology, Danvers, MA, USA), H4K16ac (ab109463, Abcam Cambridge, MA, USA), anti-RNA Pol II (sc-47701, Santa Cruz, CA, USA), anti-E-cadherin (14472 S, Cell Signaling Techology Danvers, MA, USA), anti-N-cadherin (13116 S, Cell Signaling Techology Danvers, MA, USA), anti-Histone H4 (ab177840, Abcam, Danvers, MA, USA), anti-His (PM032, MBL), anti-GST (sc-138, Santa Cruz, CA, USA), anti-GCN5 (sc-365321, Santa Cruz, CA, USA), anti-myc (M047-3, MBL), anti-α-tubulin (BE0031, EASYBIO, Beijing, China) and anti-β-actin (4967, Cell Signaling, Danvers, MA, USA) were used and the blots were developed using an enhanced chemiluminescence kit (Amersham Corp.).

### Co-immunoprecipitation (Co-IP)

After treatment, different cells were harvested and lysed in different lysis buffer to perform Co-immunoprecipitation as described before [9]. Protein A- or G-Sepharose beads (GE Healthcare, Little Chalfont, UK) were used according to the antibody used and the proteins were analyzed by Western blotting with different antibodies.

### GST pull-down assay

GST fusion proteins or His-tagged proteins were purified as described before [9]. His-tagged proteins were incubated with GST fusion proteins in TEN buffers for 4 h at 4 °C. The beads were washed three times with TEN buffers and boiled with 2X SDS loading buffer. Proteins were analyzed by Western blotting with anti-GST or anti-His antibodies and by Coomassie brilliant blue staining.

### In vitro acetylation assay

KAT8 and His-GCN5 were purified and incubated in acetylation buffer (50 mM Tris-HCl, pH 8.0, 50 mM NaCl, 4 mM MgCl_2_, 0.1 mM EDTA, 1 mM dithiothreitol (DTT), and 10% glycerol) with or without acetyl-CoA (5 mM) for 1 h at 30 °C. The reactions were stopped by adding 5 x protein sample buffer and the samples were boiled at 100 °C for 5 min before SDS-PAGE and immunoblotting.

### In vitro deacetylation assay

SIRT6 and KAT8 were purified and incubated in deacetylation buffer (10 mM Tris-HCl pH 8.0, 10 mM NaCl, 10% glycerol, 1 mM NAD^+^) for 1 h at 30 °C. The reactions were stopped by adding 5 x protein sample buffer and the samples were boiled at 100 °C for 5 min before SDS-PAGE and immunoblotting.

### Chromatin immunoprecipitation (ChIP) assay

After treatment, HCT116, SW480 or RKO cells were harvested to perform ChIP assay as described before [9]. The cross-link was reversed at 65° C overnight, and the DNA was dissolved in TE buffer and analyzed by real-time PCR. The primers for all ChIPs are available upon request.

### Cell proliferation and colony formation

For cell proliferation experiment, cells were transfected with pCMV-myc-vector, myc-KAT8 or myc-KAT8-2KR plasmids and then harvested for cell counting kit 8 (Yeasen, China) to establish a cell proliferation curve. For the colony formation assay, cells were treated as above and fixed by 4% formaldehyde and stained with 0.25% Coomassie brilliant blue after culturing normally for 2 weeks. All the experiments were performed in triplicate. The number of colonies was calculated using Photoshop (Adobe, American).

### Wound healing assay

HCT116 or RKO cells were seeded and gently scraped with a 10 μl sterile pipette tip when the cells achieved 90% confluence. The wounded cells were continuously cultured with FBS free DMEM for 2 days. The healing width were recorded at the 0 h, 24 h and 48 h under inverted Microscope. The migration rate was measured by the ratio of the day 1 and day 2 width to original width. All the experiments were performed in triplicate.

### Transwell assay

HCT116 or SW480 cells were cultured in 24-well plate with 8-μm polyethylene terephthalate membrane filters separating the lower and upper culture chambers (Corning, American). The membrane filter was coated with Matrigel (Corning, American). Then, cells were seeded and cultured by serum-free DMEM. The bottom chamber contained DMEM with 10% FBS. Cells were allowed to invade for 48 h. Before fixing, non-invasion cells on the upper side of the filter were detached using a cotton swab. Cells were fixed and stained with 0.1% crystal violet. Cells were counted in three random fields. All the experiments were performed in triplicate.

### Data analysis

Measurement data analysis was conducted using PRISM and SPSS statistical analysis software (GraphPad Software, Inc., San Diego, CA). Unpaired *t*-tests were applied to compare data between two groups, while one-way analysis of variance (ANOVA) was used to compare data among multiple groups. The statistical information of each experiment were shown in the figures and corresponding legends.

## Supplementary information

Supplemental Data

Original Data File

aj-checklist

## Data Availability

The datasets used and/or analyzed during the current study are available from the corresponding author (Yang Yang, yangsh@bjmu.edu.cn) on reasonable request.
