# Supplementary material for: KAT8 acetylation-controlled lipolysis affects the invasive and migratory potential of colorectal cancer cells
Source: Cell Death Dis. 2023 Feb 27;14(2):164. doi: 10.1038/s41419-023-05582-w (PMC9970984; doi:10.1038/s41419-023-05582-w)
Supplement: Supplementary file 1 — Supplemental Data [file 41419_2023_5582_MOESM1_ESM.pdf]

Supplemental Figure S1

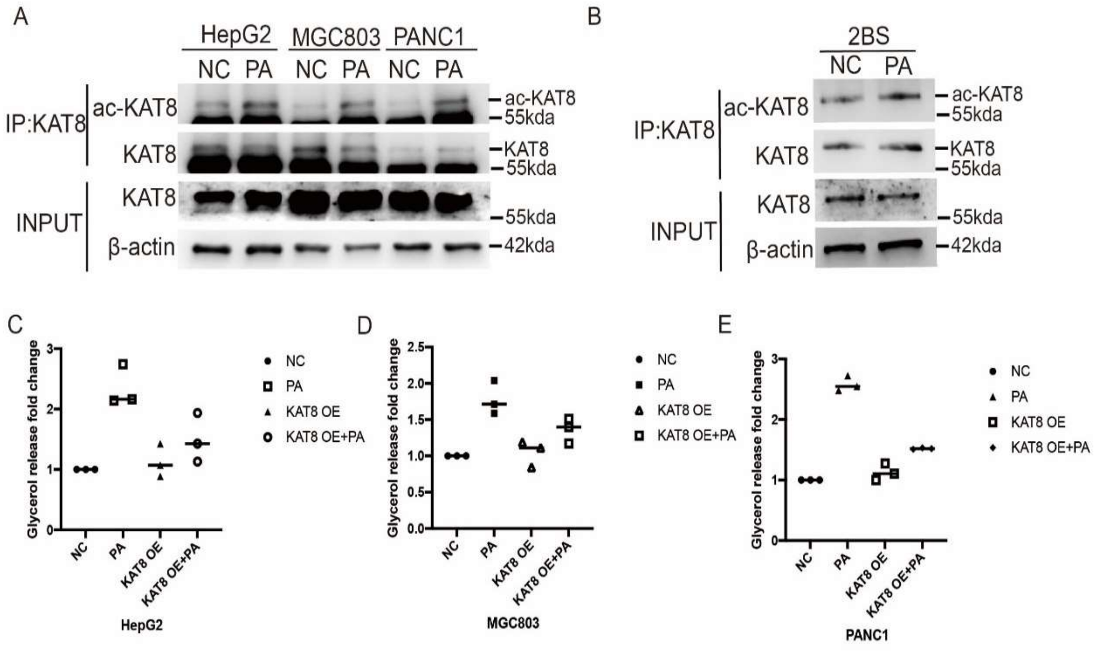

Fig S1. KAT8 regulates lipolysis accompanied by enhanced KAT8 acetylation after PA treatment in other cancer cells.

A, B HepG2 cells, MGC803 cells or PANC1 cells (A) and 2BS cells (B) were treated with or without 0.2 mM PA for 24 h, cell lysates were then extracted for a Co-IP assay to detect endogenous acetylation levels of KAT8 in these cells. **C-E** HepG2 (C) MGC803 (D) or PANC1 (E) cells were transfected with KAT8 plasmid, and then treated with or without PA at 0.2 mM for 24 h. Cells were lysed and subjected to quantify the amount of glycerol inside cells by glycerol colorimetric assay kit. The bar (−) represents the means (n =3).
