# Supplementary material for: KAT8 acetylation-controlled lipolysis affects the invasive and migratory potential of colorectal cancer cells
Source: Cell Death Dis. 2023 Feb 27;14(2):164. doi: 10.1038/s41419-023-05582-w (PMC9970984; doi:10.1038/s41419-023-05582-w)
Supplement: Supplementary file 2 — Original Data File [file 41419_2023_5582_MOESM2_ESM.pdf]

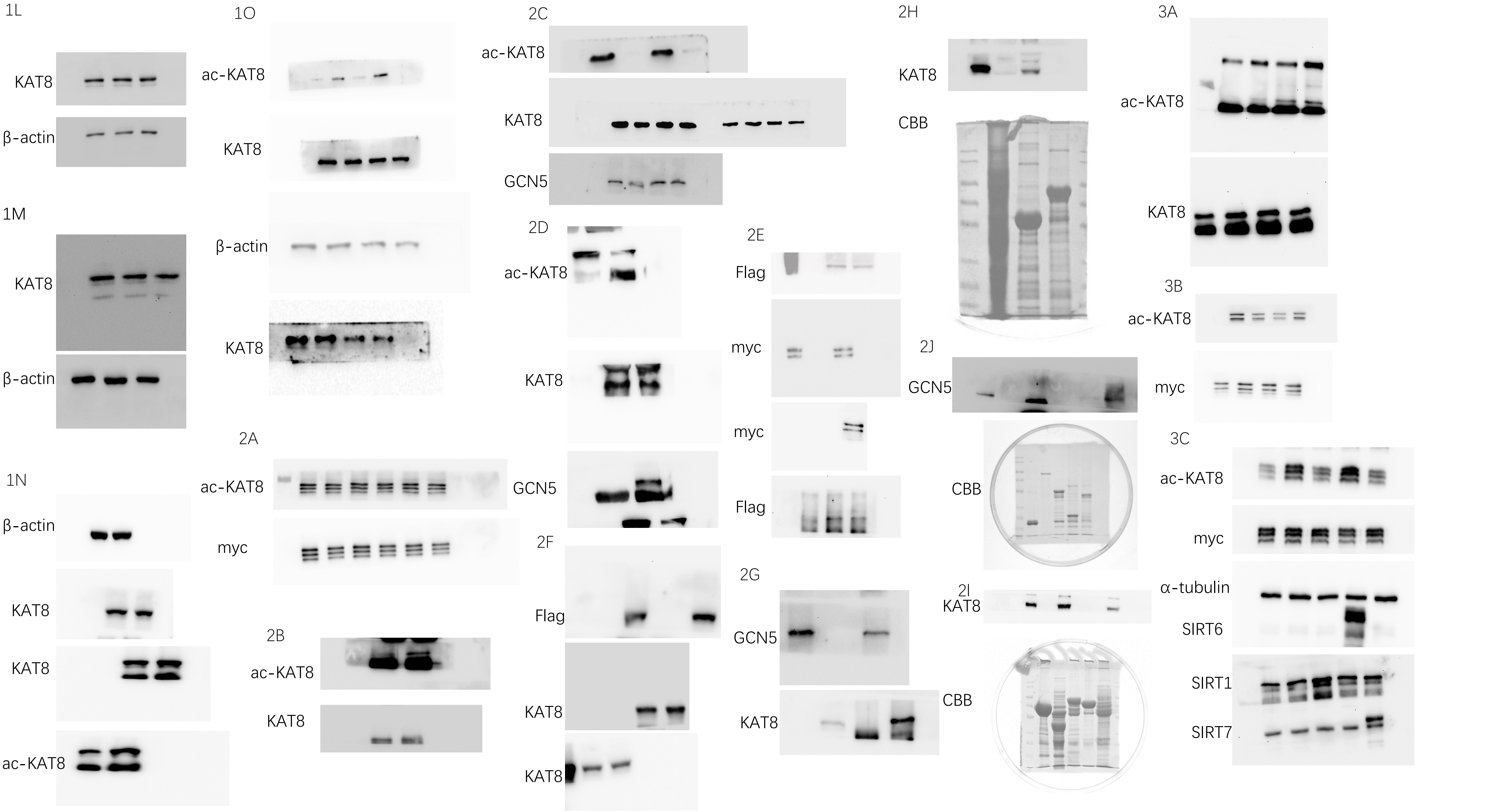
1L
KAT8
β-actin
1M
KAT8
β-actin
1N
β-actin
KAT8
KAT8
ac-KAT8
1O
ac-KAT8
KAT8
β-actin
KAT8
2A
ac-KAT8
myc
2B
ac-KAT8
KAT8
2C
ac-KAT8
KAT8
GCN5
2D
ac-KAT8
KAT8
GCN5
2F
Flag
KAT8
KAT8
2E
Flag
myc
myc
Flag
2G
GCN5
KAT8
2H
KAT8
CBB
2J
GCN5
CBB
2I
KAT8
CBB
3A
ac-KAT8
KAT8
3B
ac-KAT8
myc
3C
ac-KAT8
myc
α-tubulin
SIRT6
SIRT1
SIRT7

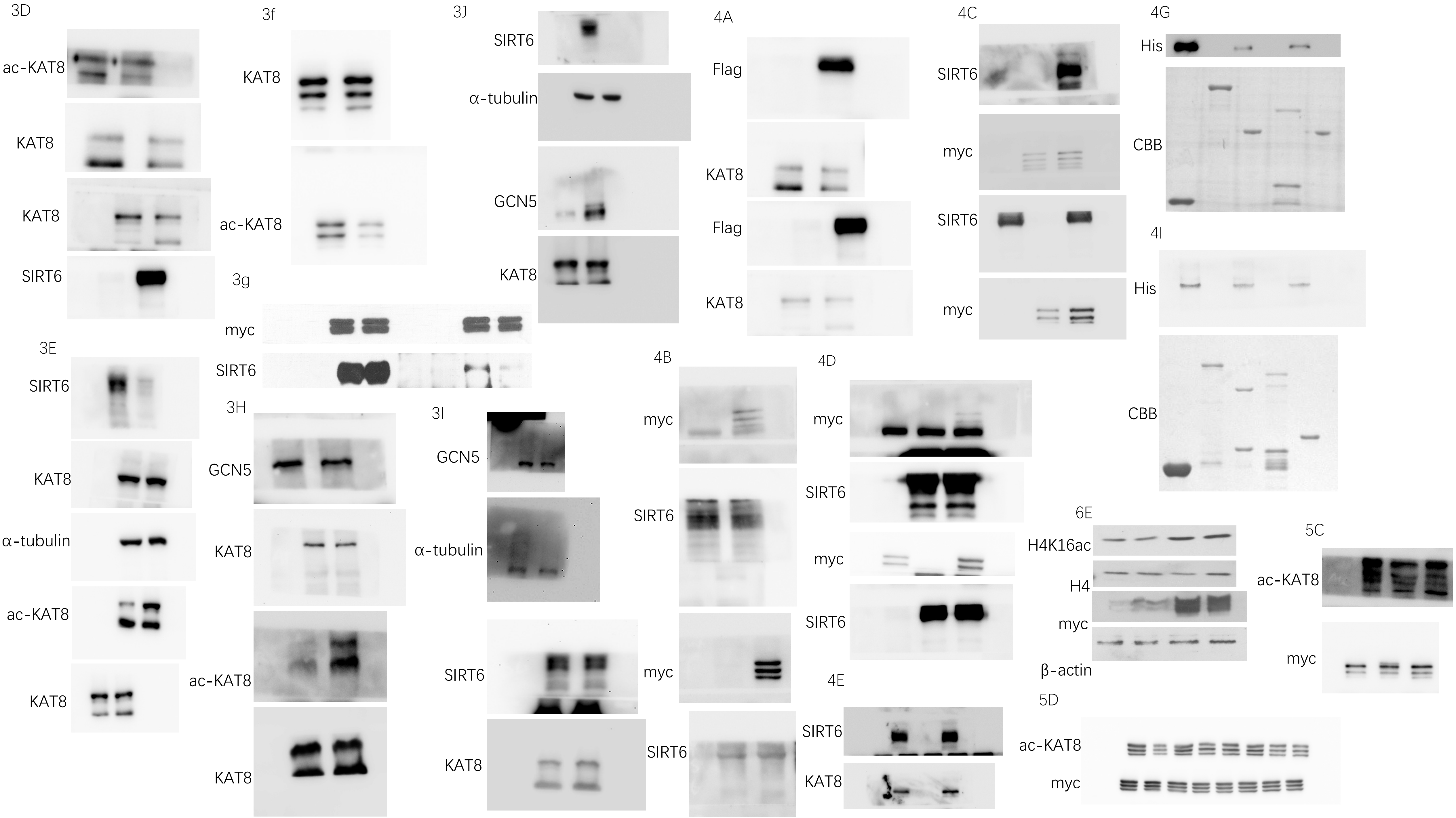
3D
ac-KAT8
KAT8
KAT8
SIRT6
3f
KAT8
ac-KAT8
3J
SIRT6
α-tubulin
GCN5
KAT8
4A
Flag
KAT8
Flag
KAT8
4C
SIRT6
myc
SIRT6
myc
4G
His
CBB
4I
His
CBB
3g
myc
SIRT6
3E
SIRT6
KAT8
α-tubulin
ac-KAT8
KAT8
3H
GCN5
KAT8
ac-KAT8
KAT8
3I
GCN5
α-tubulin
SIRT6
KAT8
4B
myc
SIRT6
myc
SIRT6
4D
myc
SIRT6
myc
SIRT6
4E
SIRT6
KAT8
6E
H4K16ac
H4
myc
β-actin
5C
ac-KAT8
myc
5D
ac-KAT8
myc

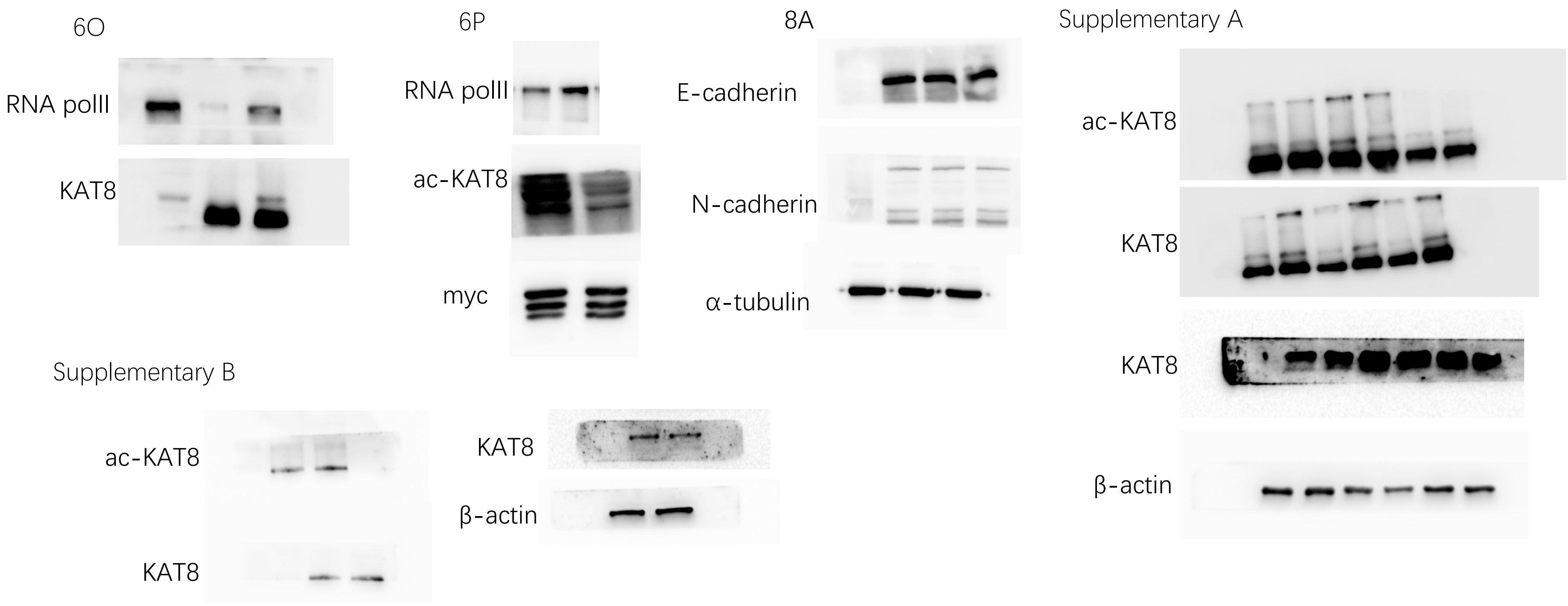
6O
RNA polII
KAT8
6P
RNA polII
ac-KAT8
myc
8A
E-cadherin
N-cadherin
α-tubulin
Supplementary A
ac-KAT8
KAT8
KAT8
β-actin
Supplementary B
ac-KAT8
KAT8
KAT8
β-actin
